# CapsNetYY1: identifying YY1-mediated chromatin loops based on a capsule network architecture

**DOI:** 10.1186/s12864-023-09217-4

**Published:** 2023-08-09

**Authors:** Zhimin Zhang, Fenglin Li, Jianping Zhao, Chunhou Zheng

**Affiliations:** 1 College of Mathematics and System Sciences, Xinjiang University, Urumqi, China; 2 Key Laboratory of Intelligent Computing and Signal Processing of Ministry of Education, Information Materials and Intelligent Sensing Laboratory of Anhui Province, and School of Artificial Intelligence, Anhui University, Hefei, China

**Keywords:** YY1-mediated chromatin loops, Capsule network, Enhancer-promoter interaction

## Abstract

**Background:**

Previous studies have identified that chromosome structure plays a very important role in gene control. The transcription factor Yin Yang 1 (YY1), a multifunctional DNA binding protein, could form a dimer to mediate chromatin loops and active enhancer-promoter interactions. The deletion of YY1 or point mutations at the YY1 binding sites significantly inhibit the enhancer-promoter interactions and affect gene expression. To date, only a few computational methods are available for identifying YY1-mediated chromatin loops.

**Results:**

We proposed a novel model named CapsNetYY1, which was based on capsule network architecture to identify whether a pair of YY1 motifs can form a chromatin loop. Firstly, we encode the DNA sequence using one-hot encoding method. Secondly, multi-scale convolution layer is used to extract local features of the sequence, and bidirectional gated recurrent unit is used to learn the features across time steps. Finally, capsule networks (convolution capsule layer and digital capsule layer) used to extract higher level features and recognize YY1-mediated chromatin loops. Compared with DeepYY1, the only prediction for YY1-mediated chromatin loops, our model CapsNetYY1 achieved the better performance on the independent datasets (AUC $> 0.99$).

**Conclusion:**

The results indicate that CapsNetYY1 is an excellent method for identifying YY1-mediated chromatin loops. We believe that the CapsNetYY1 method will be used for predictive classification of other DNA sequences.

## Introduction

By definition, each chromosome consists of a number of different chromatin domains called topological domains or topologically associating domains (TADS) [1, 2]. These chromatin domains are considered to be the basic units of chromosome folding and are regarded as important secondary structures in chromosome tissue [3, 4]. In relatively stable chromatin domains, proximal promoters and distal regulatory elements are far apart in linear distance, but the specific DNA sequences between them can interact to form a chromatin loop [5, 6].

Previous studies have shown that enhancer-promoter interaction usually occurs in the chromosomal loop structures formed by CTCF protein interaction [7, 8]. This relatively stable chromatin domain prevents enhancers from interacting with inappropriate gene promoters [9, 10]. However, normally, CTCF does not occupy these interacting elements [11, 12]. In the insulating domain formed under the action of CTCF-CTCF, Yin Yang 1 (YY1) protein binds hypomethylated DNA sequence to form homologous dimer, and then forms enhancer-promoter cytoplasmic loop structures, participating in enhancer-promoter interaction in a manner similar to the CTCF-mediated DNA cycle [13]. Barring its regulatory function in biological processes, researchers identified that YY1 may be employed as a tumorigenesis initiator. In this way, it can be used as a tumor marker for medical diagnosis and prognosis [14].

Transcription factor YY1 plays a significant role in biological activities. It is wide-range expressed in various tissues of organisms and affects cell proliferation, apoptosis, differentiation and other life processes [15]. YY1 has a complex transcriptional regulation mechanism, which can activate or inhibit the expression of different related genes. Many studies have shown that chromosome structure plays a vital role in gene regulation, but little is known about the structural interactions and mechanisms between gene promoters and their enhancers. Through reviewing the existing studies, we found that the universally expressed transcription factor YY1 promoted the enhancer-promoter structural interaction in a manner similar to CTCF-mediated DNA interaction [13]. YY1 can bind to the active enhancer and promoter elements to form a dimer, and then promotes the interaction between the related DNA elements. It has been proved that the deficiency or mutation of YY1 binding site may damage the contact between enhancer promoter and normal gene expression [13]. YY1 and CTCF have many similar characteristics. For example, they are both necessary zinc-coordination proteins that have been generally expressed. They bind to hypomethylated DNA sequences to form homologous dimers, thereby promoting the formation of dehydrogenation [16, 17]. The structure of YY1-mediated enhancer-promoter ring is similar to that of CTCF-mediated TADS, CTCF contact domain and insulation domain [18, 19]. This YY1-mediated enhancer-promoter ring structure contributes greatly to gene activation and suppression as well as to gene dysregulation in cancer.

To this day, a lot of computational methods have been used to detect chromatin interactions on the regulatory element level through genomic and CFCF binding site information in DNA loops [20, 21]. YY1 binding site information is of great significance for DNA loop recognition. Deep learning methods are available to identify YY1. For instance, Dao et al. [6] proposed a deep learning algorithm DeepYY1 based on the expression of DNA bases by word vectors, which predicted whether a pair of YY1 motifs form a loop via using the sequence characteristics of YY1 binding site [6]. Although the existing model has achieved good results, it still needs further improvement in practical application. For instance, the generalization of the model’s cross-cell prediction can be improved for better accuracy. In this paper, we proposed a new prediction model named CapsNetYY1. According to the DNA sequence of YY1 binding sites, we employed One-hot encoding method to represent the sequence features. In the aspect of feature extraction, we use multi-scale convolution layer to extract initial features of the sequence, and then a layer of convolutional layer is used to extract the fused features. A bidirectional gated recurrent unit is used to learn the feature of the CNN layer output across time steps. Finally, we use the capsule neural network for deeper feature extraction, and output prediction results. Our method achieved good performance on AUC, with AUC values exceeding 99% in both HCT116 and K562 independent datasets (testing datasets). Compared with the existing models, our computational model also shown better performance.

## Materials and methods

### Datasets

In this paper, the benchmark dataset used in the DeepYY1 [6] method was adopted. Fu-Ying Dao et al. downloaded YY1 HiChIP and YY1 ChIP-seq data from HCT116 and K562 human cells respectively [6, 13]. The dataset was defined as HiChIP chromatin loop with positive samples (probability of confidence $\ge$ 0.9), both sides of the pairing region have unique chip sequence YY1 peak, and the negative sample is defined as the confidence probability = 0. The datasets extracted 506 bp sequences centered on YY1 motif for each pair of positive and negative samples. The datasets were divided into independent datasets (testing datasets) and training datasets in a 3:7 ratio [22]. The detailed information of the dataset in this study is shown in Table 1.

**Table 1:** Details of the datasets used in this work

Cell types	Positive		Negative	
	Training	Testing	Training	Testing
HCT116	2095	898	2097	899
K562	3863	1657	3866	1657

### Feature encoding schemes

#### One-hot encoding (One-hot)

One-hot encoding is a widely used encoding method, and its application in the field of bioinformatics [23, 24] is relatively mature. In recent years, more and more studies have used heat coding to encode DNA, protein, RNA and other biological sequences, and its encoding effect has been verified in many experiments. In this study, each nucleotide in the DNA sequence is encoded into a one-dimensional digital vector with a length of 4 by One-hot encoding, as shown below: A is denoted by (1,0,0,0), C is denoted by (0,1,0,0), G is denoted by (0,0,1,0), T is denoted by (0,0,0,1). In our study, after One-hot encoding, each nucleotide sequence is encoded into a 506 * 4 two- dimensional digital matrix (DNA sequence length is 506).

### Model structure

**Fig. 1:**
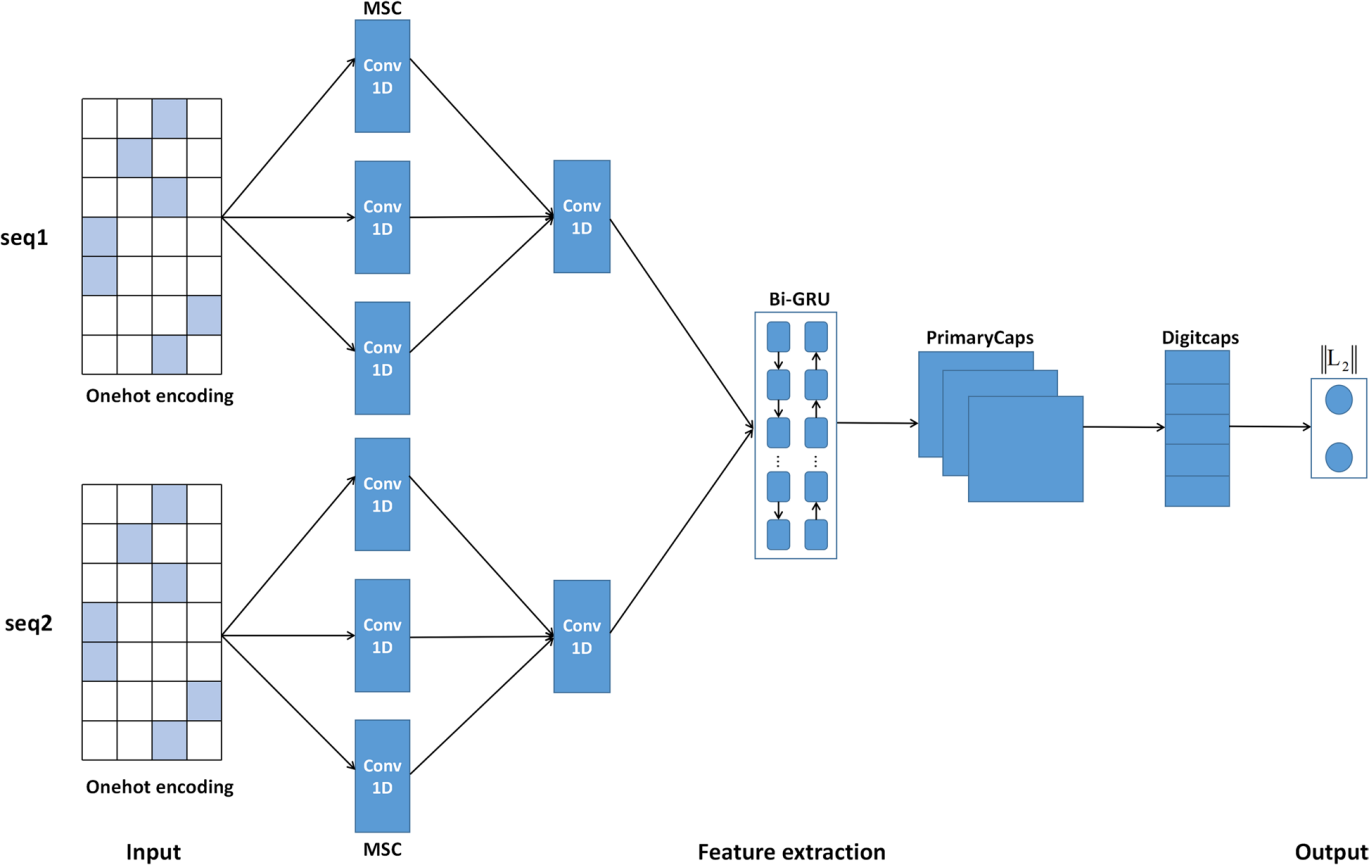
Overall architecture of CapsNetYY1

The structure of our proposed model is shown in Fig. 1. The model consists of three main steps: DNA sequence coding, feature extraction and prediction. Firstly, We used one-hot encoding to encode the DNA sequences. Secondly, multi-scale convolutional layer was used to extract initial local information features of sequences from multiple angles. Then one-dimensional convolution layer is used to extract the initial fused features and bidirectional gated recurrent unit were used to extract long-term dependence of local features of sequences. Finally, we used the primary capsule layer and the digital capsule layer to extract deeper features again, and accurate prediction and classification.

#### Convolutional Neural Network (CNN)

A growing body of literature shows that CNN is widely used bioinformatics [25, 26]. The main advantage of CNN lies in that it does not require to extract features in advance. Thus, we use the convolution layer to extract features directly from the feature encoding information of DNA sequence.

 We construct a multi-scale convolution layer, which consists of three one-dimensional convolution layers of convolution kernels of different sizes. It uses different convolution kernel sizes to convolve the feature map of some point in time and obtains new feature maps with different sizes. Then we construct a one-dimensional convolution layer to extract the fused features. In our experiment, the first convolution layer uses three convolution blocks of convolution kernels of size 3,5,7 to extract features from the One-hot encoding matrix in parallel. The three convolution blocks have 32 filters, using the relu activation function with step size 2. The second convolution layer, using a convolution kernel of size 5, 32 filters, step size 2, using the relu activation function.

#### Bidirectional gated recurrent unit (Bi-GRU)

We construct a bidirectional gated recurrent unit [27] to further extract the feature information captured by the CNN module and supplement the long-term dependency relationships in the sequence. We know that bidirectional gated recurrent units can learn long-term dependencies of local features using both forward and reverse context information. We set the output of the bidirectional gated recurrent unit to 32.

#### Capsule neural networks (CapsNet)

In the last decade, deep learnning has developed and changed rapidly. In 2017, Sabout et al. proposed a new network structure called CapsNet [28]. CapsNet is widely used in bioinformatics and has good classification and prediction effect [29, 30]. The construction of CapsNet is mainly based on capsules. Vector capsules, dynamic routing and reLu activation function are used to replace neurons, pooled operations and squash function respectively. The capsule layer of the CapsNet contains a set of neuron vectors for computing the internal information of features.

The features extracted by CNN + Bi-GRU were transported to the capsule network of one-dimensional convolution capsule layer (primary capsule layer) and digital capsule layer. Convolutional capsule layer and digital capsule layer are used for further advanced feature extraction and prediction. The convolutional capsule layer uses a one-dimensional convolutional layer, which has 16 convolutional capsule channels. Each capsule is composed of 8 convolutional units. The size of each convolutional kernel is 9 and the step size is 2. The vector capsules of the convolution capsule layer share weights with other capsules, each representing a probability. So the capsule layer requires a squeeze activation function to compress the length of each capsule between 0 and 1. The construction of the convolutional capsule layer is actually to create a dynamic routing process between the convolutional capsule layer and the digital capsule layer for the dynamic routing algorithm operation of the next layer. The digital capsule layer contains two 8-dimensional capsules, with positive capsules representing the probability of recognizing the existence of YY1-mediated chromatin loops and negative capsules representing the probability of recognizing the absence of YY1-mediated chromatin loops. Finally, the prediction results were obtained by calculating the L2 norm of the two capsule vectors.

In the capsule network, the vector length of high-level capsules represents the class probability. Thus, the high-level capsule category with the largest vector output length is selected as the model prediction category. The loss function adopted by the CapsNet is the margin loss function formula, as shown below [28]:

1 $${\mathrm{L}}_{\mathrm{k}}={\mathrm{T}}_{\mathrm{k}}\mathrm{max}{\left(0,{m}^{+}-\Vert {v}_{k}\Vert \right)}^{2}+\lambda \left(1-{T}_{k}\right)\mathrm{max}{\left(0,\Vert {v}_{k}\Vert -{m}^{-}\right)}^{2}$$

while ${\text{L}}_{k}$ represents the margin loss calculated by the above formula, ${\text{T}}_{{\text{k}}}$ is the existence value of the K th classification. If T_k_ exists, the result is 1. Otherwise, the result is 0. ${\text{m}}^{ + }$, ${\text{m}}^{ - }$, and $\lambda$, take on values 0.9, 0.1 and 0.5, respectively.

### Model training

In the present study, our models for implementing deep learning use python3.6, Keras2.1.6, and TensorFlow1.15.0. We trained each experiment using the same training strategy, and we used ten-fold cross-validation for training and independent dataset for testing. Specifically, this study used the Adam optimization algorithm [31] with a batch size of 64 and a learning rate of 0.0005 for the training. In the training iteration, our study used the early stop strategy [32, 33]. Early stop strategies are used to prevent cases of trained fitting on small datasets and to address manual epoch settings. Additionally, the parameter selection of deep learning model is also very important. The optimal of parameters is very important for the model, We adjusted the parameters according to the existing studies [34]. The parameter selection of the model is shown in Table 2.

**Table 2:** Hyper-parameters optimization

Parameters	Range	Optimal
Number of Convolution Filters	128, 64, 32,16,8	32
Convolution Kernel Size	9, 7, 5, 3, 1	3, 5, 7, 9
Bi-GRU Layer Neurons	128, 64, 32, 16	32
Capsule Dimension	16, 10, 8	8
Number of Channels	60, 32, 16	16
Number Capsule	16, 10, 8, 2	2
Number of Routings	5, 4, 3, 2, 1	3
Dropout Ratio	0.5, 0.3, 0.2	0.5
Learning rate	0.0001, 0.0005, 0.0007, 0.001	0.0005

### Evaluation metrics

In order to comprehensively evaluate the recognition performance of YY1-mediated chromatin loops, this study adopted commonly used rating indexes, including Accuracy (Acc) and F1 score. They are defined as follows [35]:

2 $$Acc=\frac{TP+TN}{TP+TN+FP+FN}$$

3 $$\mathrm{Pr}ecision=\frac{TP}{TP+FP}$$

4 $$\mathrm{R}ecall=\frac{TP}{TP+FN}$$

5 $$\mathrm{F}1=\frac{2\times \mathrm{P}recision\times \mathrm{Re}call}{\mathrm{Pr}ecision+\mathrm{Re}call}$$

In the above formula, TP and TN respectively represent the number of positive samples and negative samples correctly classified. The true label is positive samples, but all samples wrongly classified as negative samples are represented by FN. Similarly, FP represents all samples wrongly classified as positive samples while the true label is negative samples. AUC [36] refers to the area enclosed by the ROC curve. ROC curve is also called receptivity curve, which is a curve graph based on the true positive rate and false positive rate under various thresholds. AUPR [37] is the area under the precision-recall curve, which is a graph conducted drawing on precision-recall of different thresholds. Finally, F1 score [38]is calculated by the Accuracy rate and recall rate, which can be regarded as a harmonic average of the model Accuracy rate and recall rate.

## Results and discussion

### Performance evaluation on model

**Fig. 2:**
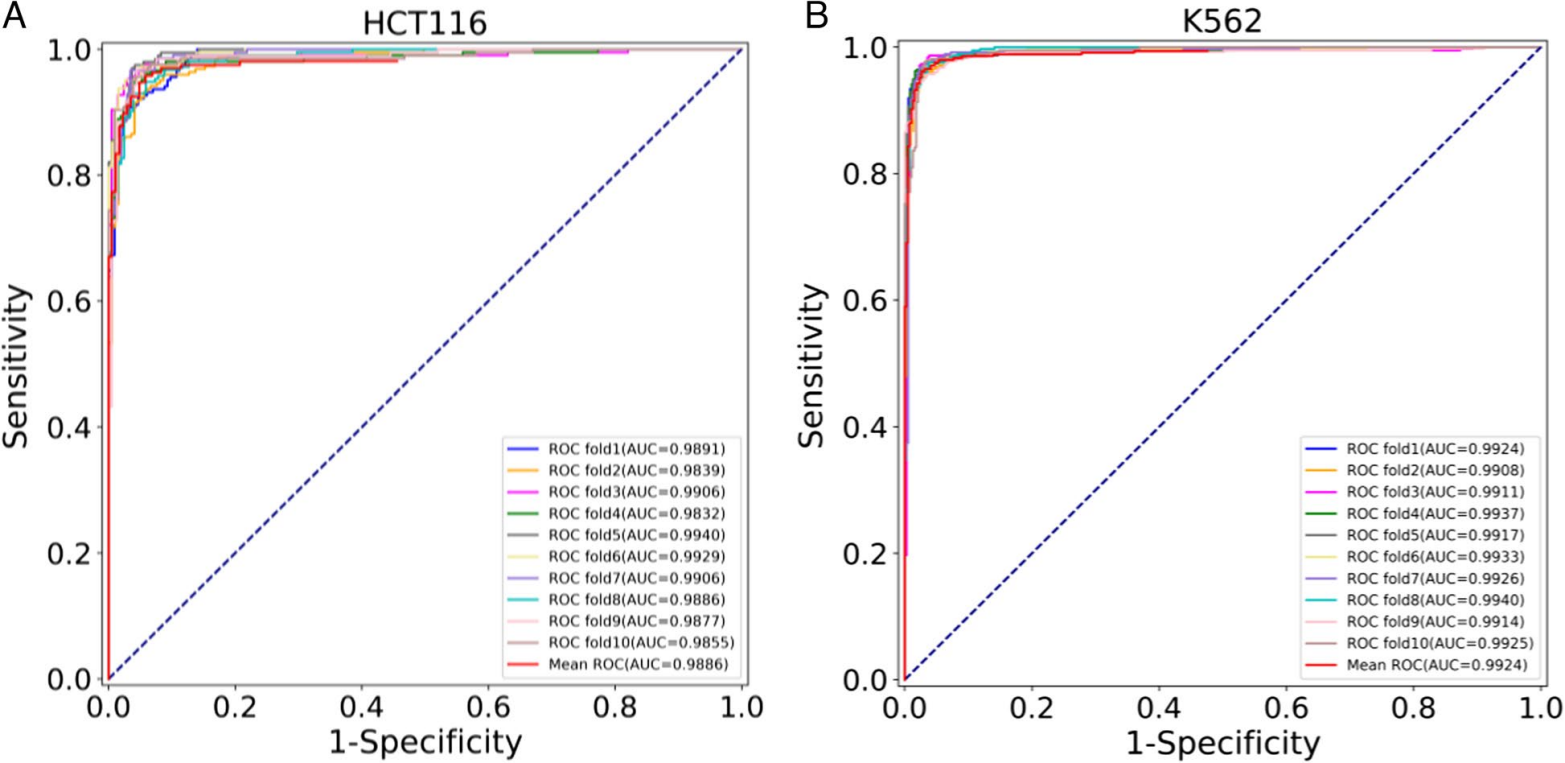
ROC curves of CapsNetYY1 models. **A** ROC curves of cell types HCT116; and **B** ROC curves of cell types K562 on the training datasets

To comprehensively evaluate the performance of YY1-mediated chromatin loops recognition, we used the same dataset as the DeepYY1 method. In order to illustrate the prediction and recognition performance of the model, we also used the ten-fold cross-validation method, and used Acc and AUC to evaluate the performance of the model. Our model achieved Acc over 0.95 and AUC over 0.98 in both cell lines through ten-fold cross-validation on the training dataset. The results were shown in Table 3, Acc and AUC of YY1-mediated chromatin loops recognition in cell line HCT116 reached 0.9544 and 0.9886. In cell type K562, the Acc and AUC of YY1-mediated chromatin loops were 0.9680 and 0.9924 respectively. These evaluation indexes can further indicate that our model has good performance in recognizing YY1-mediated chromatin loops. In addition, ROC curves of CapsnetYY1 model on training dataset of different cell types were drawn in Fig. 2 (A) and (B).

**Table 3:** Ten-fold cross-validation results

Cell type	Acc	AUC
HCT116	0.9544	0.9886
K562	0.9680	0.9924

In order to verify the robustness and reliability of the model, we will use independent datasets for verification. We used indexes such as AUC, AUPR, Acc and F1-socre to comprehensively evaluate the quality of the model. As can be seen from Table 4, both the evaluation index AUC of K562 and HCT116 cell type datasets reached above 0.99, and the evaluation index Acc and F1-socre reached above 0.95 respectively. Our model achieved the best performance on the independent datasets.

**Table 4:** Performance evaluation on independent datasets

Cell type	Acc	AUC	AUPR	F1-socre
HCT116	0.9622	0.9913	0.9917	0.9564
K562	0.9560	0.9912	0.9920	0.9583

### Ablation experiment

In order to verify the effect of our designed model structure on YY1-mediated chromatin loops prediction, we set up an experimental control group, as shown in Table 5 below. All experiments were based on the ten-fold cross-validation results under the training dataset. As can be seen from the table, when the bidirectional gated recurrent unit was removed from the model, the model performance slightly decreased. When the multi-scale convolution layer was removed from the model, Acc and AUC in HCT116 cell type datasets decreased by 1.9% and 0.55%, and Acc and AUC in K562 cell type datasets decreased by 1.19% and 0.31%, respectively. It can be seen that the multi-scale convolution layer can extract features from multiple perspectives in the sequences, thus improving the prediction and recognition effect. In summary, the bidirectional gated recurrent unit and multi-scale convolution layer in this study both of them play a positive role in the extraction of important discriminant features.

**Table 5:** Ablation experiment result

Model	Dataset	Acc	AUC
Without Bi-GRU	HCT116	0.9485	0.9874
	K562	0.9645	0.9918
Without MSC	HCT116	0.9354	0.9831
	K562	0.9561	0.9893
CapsnetYY1	HCT116	**0.9544**	**0.9886**
	K562	**0.9680**	**0.9924**

### Visualization

**Fig. 3:**
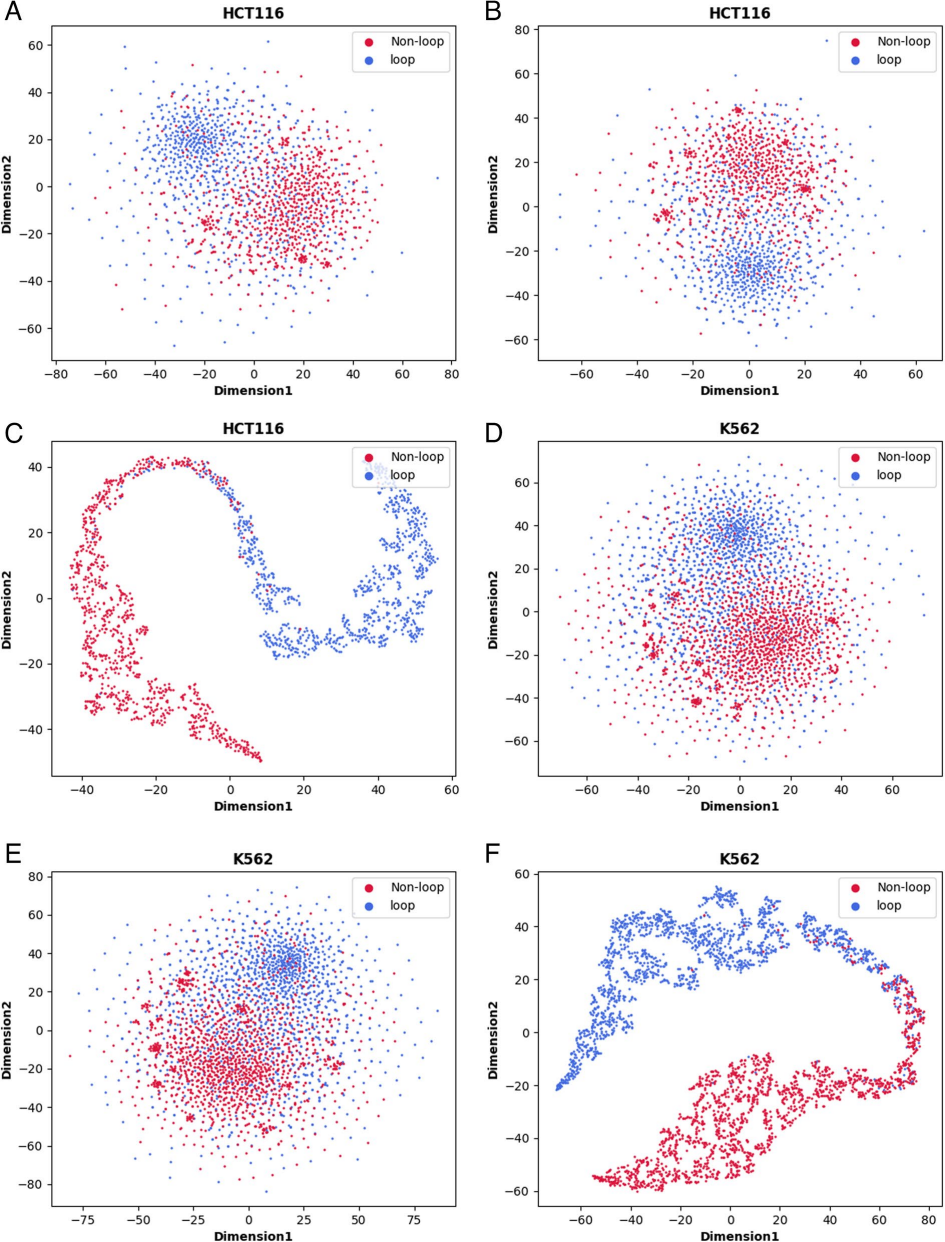
t-SNE visualization of CapsnetYY1. **A** and **B** respectively show the feature visualization after encoding of One-hot, the cell type independent datasets of HCT116. **C** represents the visualization of the results of digital capsule layer classification under the HCT116 cell type independent datasets. **D** and **E** respectively show the feature visualization after encoding of One-hot, the cell type independent datasets of K562. **F** represents the visualization of the results of digital capsule layer classification under the K562 cell type independent datasets

The CapsnetYY1 model performed well in recognizing the YY1-mediated chromatin loops, depending on the accuracy of the capsule vector captured by the capsule in the digital capsule layer. We use distributed random neighbor embedding method (t-SNE) [39] to project the high-dimensional features onto the 2-dimensional plane and intuitively display the captured discriminant features. As shown in Fig. 3, the red and blue circles respectively represent whether the corresponding labels form loops. Our model inputted two DNA sequences, so we visualized the feature visualization of one-hot encoding at input 1 and input 2 (Fig. 3A, B, D, E). Figure 3 shows the visualization results of the model on the HCT116 and K562 cell type independent datasets. It can be seen that the last layer of digital capsule layer (Fig. 3C, F) can capture features well, and whether it is loop or not is easy to separate.

### Cross cell prediction

Our model is useful for identifying chromatin loops of unknown sequences mediated by YY1. According to an existing model, DeepYY1, cross-cell prediction was verified. Thus, we also verified whether the specificity of YY1 binding site sequence was obvious between different cells. We constructed cell-specific models via using training datasets of different cell types, and then evaluated the models by adopting training datasets of two cell types as independent datasets. According to the AUC obtained in Table 6. The model based on its own dataset can achieve good performance (AUC > 0.99), while the results for other cell type datasets are still good (AUC > 0.98). This shows that our model also has a good effect on cross-cell prediction, and our model can complete cross-cell prediction. In addition, this study used the test datasets of two cell types as independent datasets to evaluate the performance of the cell-specific model as shown in Table 7. Thus, our model can identify YY1-mediated chromatin loops in another cell type using a model of one cell type.

**Table 6:** AUC values predicted across cell types under CapsNetYY1

	HCT116	K562
HCT116	0.9996	0.9859
K562	0.9884	0.9992

**Table 7:** AUC values predicted across cell types under CapsNetYY1 on independent datasets

	HCT116	K562
HCT116	0.9913	0.9884
K562	0.9881	0.9912

### Comparison with existing methods

As mentioned earlier, computational identification of YY1-mediated chromatin loops is rare, but it is also a thorny problem that needs to be solved in the field of computational biology. Therefore, we compared with the existing models to examine the validity and robustness of our model. To ensure the fairness of comparison results, we adopted the same datasets and the same evaluation indicators. As shown in Table 8, AUC values of our model in HCT116 and K562 cell types are far superior to existing model. Our model CapsNetYY1 are much better than DeepYY1 model. The AUC value of CapsNetYY1 in HCT116 cell types was 4.28% higher than that of DeepYY1. The AUC value of K562 cell type was 5.74% higher than that of DeepYY1. The results manifest the importance of our model in identifying YY1-mediated chromatin loops.

**Table 8:** Comparison with existing methods in independent datasets for the values of AUC

Method	HCT116	K562
DeepYY1	0.9485	0.9338
CapsNetYY1	**0.9913**	**0.9912**

## Conclusion

In this study, we proposed a new computational model (CapsNetYY1). This is a capsule network based on multi-scale convolution and bidirectional gated recurrent unit for the recognition of YY1-mediated chromatin loops. The results from independent datasets shown that CapsNetYY1 can predict YY1-mediate chromatin loops with good performance. CapsNetYY1 model is more effective in recognizing YY1-mediated chromatin loops than the current advanced methods, and has better generalization and robustness. This makes CapsNetYY1 a potential tool for solving other DNA sequence prediction tasks.

## Data Availability

The data used to support the findings of this study are available from JASPAR (ID:MA0095.1) and online from http://lin-group.cn/server/DeepYY1/download.html. The source code is available at https://github.com/zhangzhimin1108/CapsNetYY1.

## Abbreviations

YY1: Yin Yang 1
DeepYY1: A deep learning approach to identify YY1-mediated chromatin loops
CapsNet: Capsule neural networks
CNN: Convolutional neural network
AUC: Area under the ROC curve
ROC: Receiver operating characteristic
PR: Precision-recall curve
AUPR: Area under the precision-recall curve
